# Host–Pathogen Interaction Interface: Promising Candidate Targets for Vaccine-Induced Protective and Memory Immune Responses

**DOI:** 10.3390/vaccines13040418

**Published:** 2025-04-16

**Authors:** Gloria G. Guerrero, Vicente Madrid-Marina, Aurora Martínez-Romero, Kirvis Torres-Poveda, Juan Manuel Favela-Hernández

**Affiliations:** 1 Unidad Académica de Ciencias Biológicas, Universidad Autónoma de Zacatecas, Zacatecas 98600, Zac., Mexico; 2 Centro de Investigación en Enfermedades infecciosas (CISEI), Instituto Nacional de Salud Pública (INSP), Cuernavaca 62100, Mor., Mexico; 3 Facultad de Química, Universidad Juárez del Estado de Durango, Gómez Palacio 34100, Dgo., Mexico; 4 Secretaria de Ciencia, Humanidades y Tecnologías (SECIHTI), Instituto Nacional de Salud Pública, Cuernavaca 62100, Mor., Mexico; 5 Instituto Multidisciplinario de Ciencias “Avicena”, Torreón 27250, Coah., Mexico

**Keywords:** Gram-positive, Gram-negative pathogenic bacteria, vaccines, adjuvants, innate, cellular immune response, prime boost, route of immunization

## Abstract

Vaccine formulations are a successful strategy against pathogen transmission because vaccine candidates induce effective and long-lasting memory immune responses (B and CD4+ T cells) at systemic and mucosal sites. Extracellular vesicles of lipoproteins, bioactive compounds from plants and invertebrates (sponges) encapsulated in liposomes, and glycoproteins can target these sites. The vaccine candidates developed can mimic microbial pathogens in a way that successfully links the innate and adaptive immune responses. In addition, vaccines plus adjuvants promote and maintain an inflammatory response. In this review, we aimed to identify the host–pathogen interface as a rich source of candidate targets for vaccine-induced protective and long-lasting memory immune responses.

## 1. Introduction

The list of infectious diseases caused by intracellular pathogens is long [1,2], while the number of vaccines developed against them is much shorter. Historically, vaccines have been immunology’s most outstanding contribution to the fight against serious infectious diseases caused by bacteria, viruses, parasites, fungi, and even non-infectious diseases, autoimmune, or neurodegenerative diseases [1,2,3,4,5,6,7]. The history of vaccination goes back to the first dated vaccination with a live whole organism by Edward Jenner in the 18th century, and, since then, the development and production of vaccines has gone through several stages with promising results [3,4,5,8,9,10,11]. The development of vaccines has involved the use of whole organisms (poxvirus, etiological agent of smallpox (Edward Jenner in the 18th century)), attenuated organisms (*Mycobacterium bovis Bacillus Calmette-Guérin*, Louis Pasteur), or attenuation in vivo (animals, (anthrax, rabies, 19th century). With the advancement and application of molecular biology and genetic engineering, it has been possible to develop vaccines based on the various microbial and viral components. Plotkin S [1] has recently reviewed a list of vaccines currently licensed, and many other studies and reports have reviewed vaccine development [2,3,4,5]. Vaccine development in the 21st century is moving from the natural to the whole organism, through attenuation and inactivation by physical means or through animal or cell culture passages or by using various biotechnological tools, such as omics technologies [1,2] to gain insight into the most optimal vaccine formulations [9,10].

**Figure 1:**
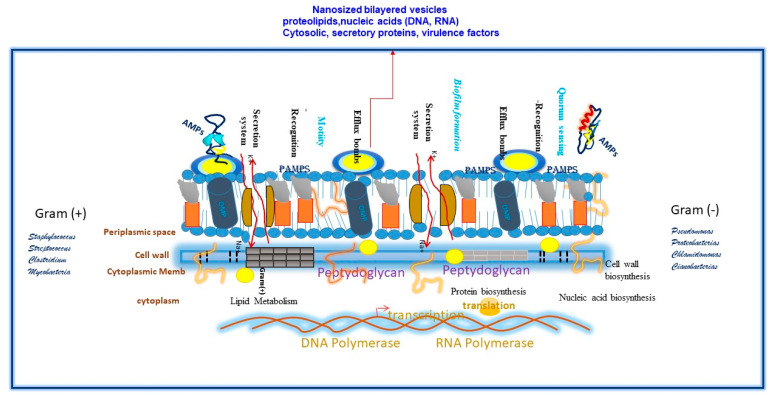
The membrane surface of Gram-positive and Gram-negative bacteria is composed of a number of molecular components that shape the interaction with the host. Lipopolysaccharides (LPS), peptidoglycans, mycolic acids, and toxins are known as pathogen-associated molecular patterns or PAMPs. Transmembrane pumping systems, such as porins and the secretory two-system component, allow bacteria to secrete antimicrobial peptides, all of which are potential vaccine candidates whose design, based on nanotechnology and various multi-omics technologies, opens up the range of possibilities from genome mining to the optimization of even artificial intelligence to predict candidate vaccine formulations for the induction of long-lasting memory and protective immune responses.

To advance the vaccine development against microbial pathogens, it is pivotal to dissect the molecular components that are immunodominant from the whole organism as they represent the signature of the host-pathogen interaction [12]. For example, carbohydrate-based vaccines have emerged as one of the most promising subunit vaccine candidates. Because carbohydrates and glycol conjugates are involved in several biological processes, including host-pathogen interactions; cell communication; proliferation and differentiation; and the initiation of immune responses, proteins and carbohydrates enhance the immunogenicity of vaccines. The immunogenicity of carbohydrates is based on different cell surface epitopes characteristic of bacterial pathogens, including bacteria and viruses [13]. Bacterial glycan patterns, which differ from mammalian cells, show higher conservation of pathogen serotypes than protein components [14]. In addition, carbohydrate immunogenicity is related to the structure of carbohydrate epitopes and alternative vaccine antigen carrier systems. The glycan antigens for CPS-based glyco-conjugate vaccines are mainly obtained from bacterial fermentation and conjugated to carrier proteins to form the so-called homogeneous fully synthetic glyco-conjugate vaccines [15]. The pneumococcal polysaccharides are weakly antigenic; a protein conjugated to capsular polysaccharide vaccines has helped to overcome the weak immunogenicity of pneumococcal polysaccharides and reduced the incidence of pneumococcal disease, especially in the pediatric population [16]. Another bacterial pathogen, *Escherichia coli* O148, is a non-encapsulated enterotoxigenic (ETEC) Gram-negative bacterium that can cause diarrhea, hemorrhagic colitis, and hemolytic uremic syndrome in humans. The surface-exposed O-specific polysaccharide (O-SP) of the lipopolysaccharide of this bacterium is both a virulence factor and a protective antigen. It is composed of the linear tetrasaccharide repeating unit [3]-α-L-Rhap-(1 → 2)-α-D-Glcp-(1 → 3)-α-D-GlcNAcp-(1 → 3)-α-L-Rhap-(1→) and differs from the O-SP of *Shigella dysenteriae* type 1 (SD) only in that the latter contains a D-Galp residue instead of the glucose moiety of the former [17]. In addition, the use of liposomes and exo-vesicles to target systemic and mucosal compartments mimics the action of living organisms as much as possible, resulting in the initial interaction between the pathogen-associated molecular pattern (PAMPs) and the pattern of receptor recognition (PRRS) on antigen-presenting cells (APCs) (macrophages, dendritic cells) [6,18,19] (Figure 1). The more specific the vaccine formulation, the better the targeting of inductive and mucosal sites for activation and the induction of CD4+ and CD8+ T cells and B lymphocytes for the production of optimal levels of secretory IgA and subclasses of IgG antibodies [18,20,21]. These characteristics strongly influence the nature and magnitude of humoral and cellular immune responses [5]. For example, conjugated vaccines have been reported to induce a T-cell-dependent response involving the interaction of specialized CD4+ T cells, called follicular helper T cells (Tfh), with the germinal center of B cells in secondary lymphoid organs [5]. Furthermore, the contribution of nanotechnology to the formulation of vaccine candidates is now one of the more promising alternatives to target local sites and to reach tissue-resident T cells, and a way of tuning (immunomodulation) the inflammatory response necessary to prime the host immune system in both trained innate and adaptive immune memory responses [1,2,3,4,5,9,10,11,20,21]. One example is the development of vaccine formulations in nano-hybrids, such as OMV.SIRPakoha@CaP/GM-CSF, which consists of bacterial components and which is targeted to exert vaccine-enhanced anti-tumor activity. Despite some technical issues, nano-hybrids consisting of bacterial outer membrane vesicles (OMVs) contain an abundance of PAMPs and have the potential to act as TLrV inducers and tumor-associated macrophages (TAM) that mediate anti-tumor phagocytosis [19].

**Figure 2:**
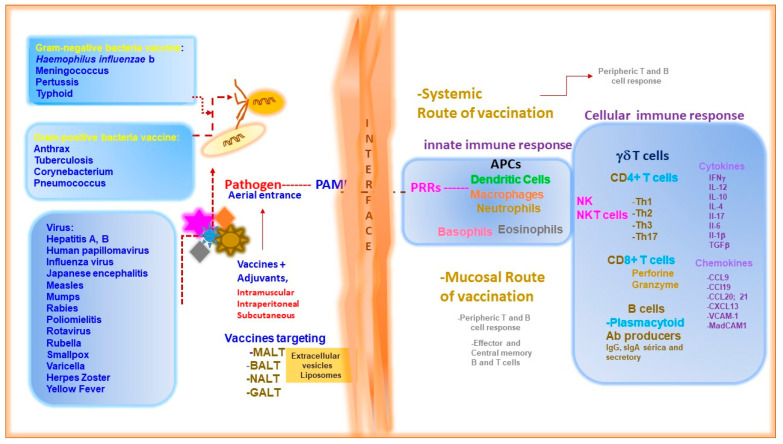
Schematic representation of the host–pathogen interaction interface. The interaction at the interface is between the pathogen and the host and the interaction is mediated by the pathogen-associated molecular patterns (PAMPS) and the patterns of receptor recognition (PRRs) on the surface of antigen-presenting cells (APCs). The vast majority of pathogens are airborne. When an antigen enters the body by the intramuscular/subcutaneous route, it reaches the inductive sites in the lymph nodes and elicits a systemic immune response, but does not activate the mucosal immune system. However, if the antigen enters via the nasal/oral route, it reaches the inductive sites in the gut and there are two possibilities: (1) the induction of a systemic immune response and (2) a mucosal cellular immune response. There is also induction of chemokines and cytokines and the expression of homing receptors. The vaccine candidates mimic the pathogens and follow a similar pathway for the induction of systemic or mucosal protective and immune responses, depending on the formulations. Vaccines targeting one of the mucosal compartments (BALT, NALT, MALT, and GALT) require a particulate formulation, such as exo-vesicles, exosomes, liposomes, plus the adjuvant. Current vaccines against microbial or viral pathogens are listed on the left.

Thus, basic and applied research has progressively developed strategies against several infectious agents (Figure 1). In this context, the tools with this purpose, molecular biology, microbial genetics, reverse genetics (the modification of the activity of a target gene to analyze the phenotypic consequences, approached through mutations, RNAi) [22,23,24], genetic engineering, and, more recently, omics technologies (metagenomics, genomics, metabolomics, proteomics, and transcriptomics). Specifically, one of the genetic techniques that has contributed to this progress in vaccine development is reverse genetics. Several types of mutants are generated, for example: (a) mutants generated by adaptation to cell culture (e.g., measles, oral polio vaccine, varicella, and mumps), (b) cold-adapted mutants, and (c) autotrophs from Salmonella enteric (Ty21a) [25] and *Mycobacterium tuberculosis* [26] or extracellular vesicles [10,27,28,29]. Therefore, in this review, we aimed to pinpoint the host–pathogen interaction interface as a rich source of molecular components that are potential candidates for targeting effector and protective immune responses in the systemic and mucosal compartments (Figure 2) with implications for therapeutic and vaccine development [1,2,4,5,9,10,18].

## 2. Host–Pathogen Interaction Interface

The host–pathogen interaction interface begins at the physicochemical surface of the skin and the mucosal tissues of the gut and the various tracts [18,20]. The physicochemical composition of the skin and mucosal tissues, e.g., the mucosa, which is mainly composed of mucins and mucus, and the sweat and sebaceous glands, which secrete urea, amino acids, and nutrients for bacteria and the like, can cross this first physical barrier [6,11,18,19,20,21]. At the surface of the skin or mucosal barriers, pathogens interact specifically with epithelial and M cells in the mucosal tissues, leading to various events, such as intercellular communication through the expression of chemokines CCL9 and CCL20 to promote the recruitment of dendritic cells to the associated follicles or Peyer’s patches, resulting in antigen presentation, inflammatory reactions, and immune metabolic responses [4,19,27,28,29]. In terms of vaccination, one of the most important outcomes of the interaction between the vaccine (antigen) and host (immune system) is the activation differentiation of B and T cells [4,5,6,9,12,18,19,20,21,30,31] (Figure 2). Another consequence of the interaction between host pathogens (in the case of protozoa) that cause disease in humans, such as the protozoan pathogens, is that they can phagocytose or engulf T cells and cross-dress molecular components (an immune or non-immune cell acquires peptides, antigens, or MHC molecules from a donor cell) of the pathogen and host cells [32,33]. Who is who at the interface? [34,35,36]. On their own, pathogens and their PAMPs (dsRNAs, dsDNAs, high methylated cytosine phosphate guanosine CpG), glycolipids, lipids, proteins) are recognized by PRRS (a set of different receptors, Toll-like receptors, RIG-like receptors, NOD-like receptors). Upon interaction, the cross-talk initiates signaling cascades that stimulate the induction of interferon (IFN) expression [11,37,38,39,40].

These secreted cytokines induce the expression of IFN-stimulated genes (ISGs) that encode antiviral and anti-microbial factors, enabling other cells to mount an anti-pathogen response [41,42]. Some ISGs encode members of the ADP-ribosyl transferase (ART) family. In particular, the mono-ADP transferases are responsible for regulating the antiviral immune response. ART and MADP function to regulate either the host or the viral immune response [11,43]. Cellular biological processes such as phagocytosis, endocytosis and trogocytosis [32,33], in addition to triggering different signaling pathways, favor one side of the control of the pathogen or the establishment of the pathogen (Figure 2). Interaction with the membrane receptor on antigen presenting cells, immune, and non-immune cells leads to an internalization process in which the cytoskeleton, tubulin, and clathrin system may be involved [32,33]. Some pathogens undergo a process of endocytosis that results in structural changes, ranging from activation of the mydosome, inhibition of autophagy and proteasome, and MHC presentation to naive CD4+ T cells [36,37,38], to regulation of chromatin by some pathogens. On the other hand, metabolic pathways affected by host–pathogen interaction include the eicosanoid-prostaglandin network. This pathway is associated with pro-inflammatory cytokines that limit the increase of type I IFNs [11,12,39,40,41]. At this point, some drugs can manipulate these networks by modulating the host immune response and preventing microbial infections [34,37,39,40,41].

Furthermore, the host–pathogen interaction at the interface of innate and non-innate cells has several outcomes: several bacterial or microbial components, and even viruses, are chemically enabled to play key roles in the host–pathogen interaction, resulting in immunomodulation, pathogenesis, and symbiosis [4,6,9,11,21,33,34,41,42]. How it happens and where it starts: As discussed above, the most classical and cosmopolitan interaction is between the PAMPs (patterns associated with molecular to pathogens) and the PRRS (patterns of recognizing receptors), establishing cross-talk primarily at the interface of the membrane surface on antigen-presenting cells, epithelial cells, neutrophils, and leucocytes [12,36,37,38,39,40]. The first, represented by the molecular components of both types of bacteria, relates to virulence factors, such as toxins, efflux pumps, glycoproteins, adhesion, secretion products, and molecular components of dormancy and lipids. The second is the set of different receptors on the membrane surface, such as Toll-like and lectin-like receptors, and in the cytoplasm, such as NOD, Toll-like receptors (TLR9, TLR13) and RIG-I receptors [36,37,38,39,40]. Interestingly, cells (i.e., bacteria) or microbial components act as effectors, exerting an effect at the nuclear level to modulate host chromatin. Intracellular pathogens, such as *M. tuberculosis,* can induce cross-talk involving arachidonic acid, the COX system, and the pro-inflammatory IL-1b [3,12,42,43,44]. Another example is the proteobacterium, an enterobacterium that can solubilize hydrophobic compounds, representing a potential carrier of drugs or chemicals, or even being used in nanotechnology, as is the case of the cyclic β-1, 2-D-glucans (CβG)], a bio-nano polymer composed of sugar rings of 17 to 25 D-glucose units linked exclusively by β-1, 2-glycoside linkages [45,46], which has immunomodulatory properties and has been proposed as a novel adjuvant for vaccine development. A third example of cross-talk involves the induction and activation of ADP-ribose phosphatases [40]. Inflammation leads to the induction of extracellular traps (NETs). These NETs are made up of microbial networks of chromatin, proteins, and oxidative enzymes [47,48], providing an avenue for manipulating neutrophils to induce NETs. The regulation of chromatin by microbial effectors to take control of key host cellular functions, such as the cell cycle, and leave their transcriptional signature allows the identification of therapeutic targets [47,48] (Figure 2).

## 3. Microbial Components for the Development of Vaccines

The microbial molecular components are the outer membranes, cell wall, and inner membranes, as well as the proteins involved in the secretion and transport system and/or proteins involved in adhesion and dissemination of bacteria, for example, the mycolic acids and glycolipids of the mycobacterial cell envelope of pathogenic mycobacteria belonging to the *M*. *tuberculosis* complex [49,50,51,52]. The lipoteichoic acid (LTA) in the cell wall of Gram-negative bacteria, a polymer of alternating units of a polyhydroxylalkane containing glycerol, ribitol, and phosphoric acid, are linked to form phosphodiester units very similar to those found in the periplasmic glucans in the envelope of Gram-positive bacteria [52]. Another example is the virulence factors of *H. pylori* that contribute to gastric disease, such as recombinant *H. pylori* urease co-administered with native *Escherichia coli* (*E. coli*) enterotoxin (LT), which has been shown to reduce the *H. pylori* load in infected individuals. In addition, the use of selected antigens known to be involved in the pathogenesis of infection, such as urease, vacuolating cytotoxin (VacA), cytotoxin-associated antigen (CagA), neutrophil-activating protein (NAP), and others, may confer prophylactic or therapeutic protection in animal models [53].

As the problem of *Helicobacter pylori* (*H. pylori*) infection is the development of antibiotic resistance, the main types of vaccines for *H. pylori* have been pursued, based on whole bacterial vaccines, vector vaccines, subunit vaccines, nucleic acid vaccines, and epitope vaccines. Of importance is the optimization of the oral therapeutic *H. pylori* vaccine with improved immunogenicity of the virulence factors UreA and UreB and long-term immunity [54], which plays a key role in the design and development of *H. pylori* vaccines. At present, a multivalent epitope vaccine FVpE against four key *H. pylori* virulence factors (urease, CagA, VacA, and NAP) and oral immunization with the FVpE vaccine plus a polysaccharide adjuvant (PA) containing *Lycium barbarum* polysaccharide and chitosan could protect against *H. pylori* infection in the Mongolian gerbil model. Indeed, most of the candidate antigens used as *H. pylori* vaccines have shown good immune protection in animal studies; however, only a few vaccines perform well in clinical trials, as highlighted by several reports in the literature, including whole-cell antigens and virulence antigens, such as UreB, VacA, CagA, and HspA. The only *H. pylori* vaccine in phase 3 clinical trials is a recombinant subunit vaccine consisting of urease subunit B (UreB) as the prophylactic vaccine antigen. Classical mucosal adjuvants such as cholera toxin (CT) and heat-labile enterotoxin (LT) from *E. coli* and other mucosal adjuvants are used to combine each of these components in the vaccine. In addition, vaccine(s) against *H. pylori* will depend on either the immunogenicity of exosome vaccines and/or the use of the full range of mucosal adjuvants for systemic and mucosal immune responses [55]. For other Gram-positive pathogens, such as *Staphylococcus aureus (S. aureus)*, one of the most common human pathogens worldwide, the high antibiotic resistance profile has prompted the search for novel interventions, such as vaccines, rather than new antibiotics. The 13-valent pneumococcal polysaccharide conjugate vaccine (PCV13) protects against lethal infection by *S. pneumoniae,* a major invasive Gram-positive pathogen of human community-acquired infections. This conjugate vaccine (PCV13) has been consistently associated with serotype-specific immune protection in animal models. Several new vaccine candidates based on bacteriophages, monoclonal antibodies, centirins, and new classes of antibiotics are being evaluated in clinical trials in target populations [56]. Furthermore, the candidates’ reliability and sensitivity correlate with vaccine efficacy, as demonstrated with whole-cell vaccines against *Klebsiella pneumoniae* and *E.coli*, two important Gram-negative pathogens in hospital-acquired invasive infections. This system can be used as an effective readout to assess the immunoprotective potential of vaccine candidates in the preclinical phase, bridging the current technical gap in vaccine evaluation between conventional in vitro approaches (antibody production and pathogen neutralization/opsonophagocytosis) and the survival of immunized animals [57]. Another immunogenic component targeted for vaccine development is trehalose, a disaccharide of two D-glucose molecules that plays structural and functional roles in the mycobacterial cell envelope [58,59,60]. The mechanism of action of trehalose and/or trehalose derivatives is host colonization and growth and it may modulate interactions with host defense mechanisms [58]. In addition, trehalose protects against abiotic stress and may be a target in several bacterial vaccines. More recently, there has been interest in the role of trehalose or trehalose derivatives in the microbial virulence of Gram-positive and Gram-negative animal and plant pathogens. Moreover, Gram-positive and Gram-negative bacteria can release nano-sized lipid bilayer structures called membrane vesicles (MVs). These MVs play a role in bacterial survival by orchestrating interactions between bacteria and between bacteria and the host [61]. Indeed, extracellular vesicles from cells of all types of organisms, including Gram-negative and Gram-positive bacteria, are outer membrane vesicles (OMVs) or membrane vesicles (MVs) released as extracellular lipid bilayer-derived particles that can induce inflammatory responses to specific pathogens, such as *S. pneumoniae*, *P. aeruginosa*, and *L. pneumophila*. Initial studies have demonstrated the efficacy of such vaccines in animal models exposed to (O) MVs from *B. pertussis*, *S. pneumoniae*, *A. baumannii*, and *K. pneumoniae* [62]. Moreover, OMVs mediate bacterial virulence in pneumonia by challenging the host’s respiratory epithelium and cellular and humoral immunity, which enables their natural antigenicity and favorable biochemical properties. Of note is that the secretion system of *Mycoplasma* spp produces and releases several components into its environment, including polypeptides, exopolysaccharides, and extracellular vesicles, and it can be considered part of the mycoplasma releasome and its role in the interaction of mycoplasmas with host cells and tissues [63]. In summary, two important microbial components of the physiology and virulence factors that can be targeted for the development of vaccine development are the following: (1) bacterial secretome, which consists of soluble virulence mediators and a significant proportion of extracellular vesicles—lipid bilayer-bound particles that are integral mediators of intercellular communication and (2) the bacterial releasome of Gram-positive and Gram-negative membrane vesicles which comprises polypeptides expressed at the cell surface or released into the extracellular environment, defined as bilayer proteolipids enriched in bioactive proteins, lipids, nucleic acids, and virulence factors [64]. On the other hand, referring specifically to the development of vaccines for virus infections, vaccines against COVID-19, are of the first generation, represented by mRNA, viral vector, and whole inactivated virus vaccines. There is a combination of first-, second-, and third-generation mRNA vaccines against zoonotic influenza viruses, such as avian influenza A/H5N1 and A/H7N9 [65]. FLUCOV-10 is a novel 10-valent second- and third-generation mRNA vaccine derived from a platform encoding hemagglutinin (HA) proteins from four seasonal and two avian influenza viruses and SARS-CoV2. The novel mRNA-based combination vaccine contains genes for surface glycoproteins from different influenza viruses and SARS-CoV-2 variants and is recognized as a potential and unique adaptable vaccine. Thus, the respiratory syndrome coronavirus-2 spike (S) protein-based vaccine candidate is one of the candidate antigens with the most potential for the second- and third-generation vaccines using modern platforms [64]. Zhang et al., 2022 [65] reported a mosaic-type trimeric form of the spike receptor-binding domain (mos-tri-RBD) as a broad-spectrum vaccine candidate carrying key mutations from Omicron and other circulating variants. Interestingly, a vaccine combining molecular components from different organisms has been developed, such as the intranasal COVID-19 subunit vaccine. This is a recombinant six-proline-stabilized D614G spike protein (mC spike) from the SARS-CoV-2 vaccine linked to *Neisseria meningitidis* outer membrane vesicles (OMVs) via the LPS-binding peptide sequence mCramp (mC)

## 4. Vaccine-Induced Immunological Memory

**Figure 3:**
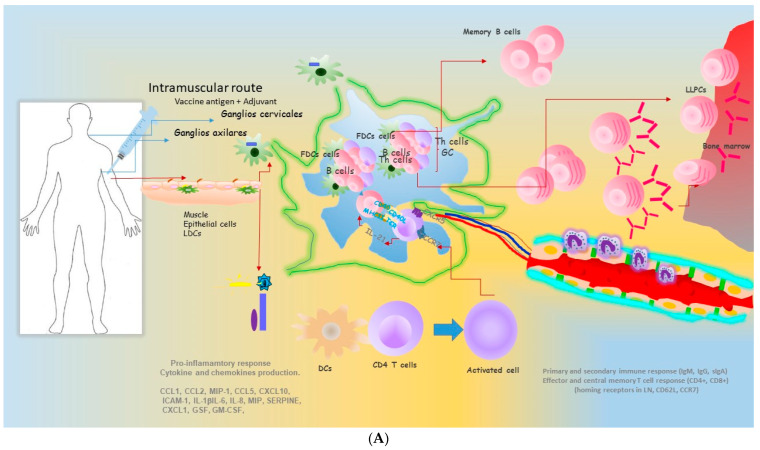

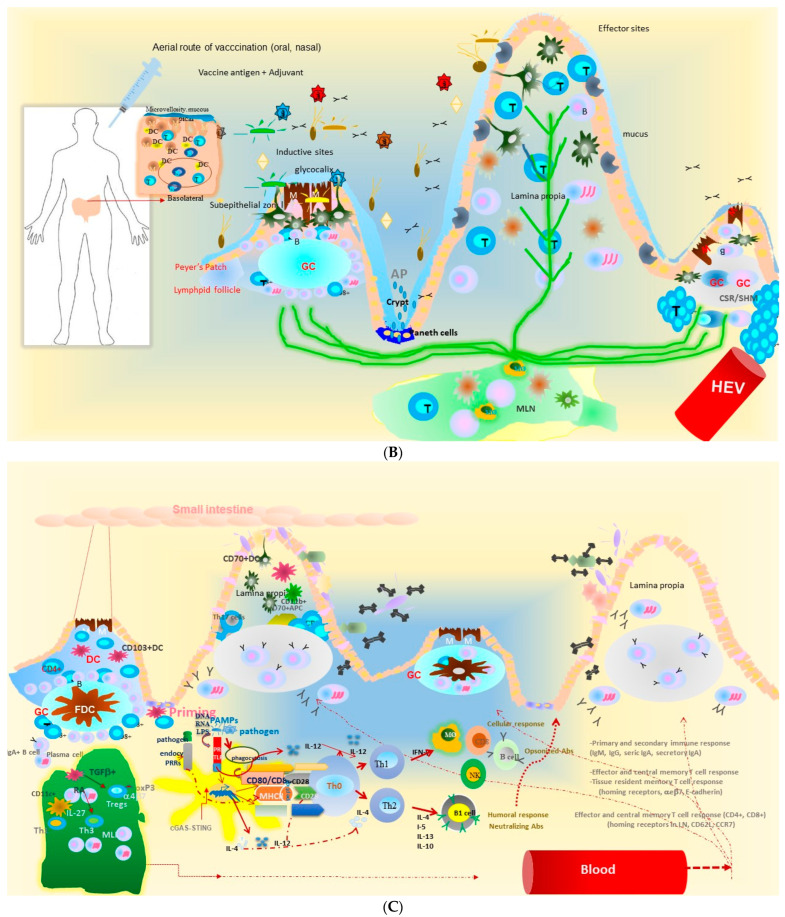
Vaccine induced protective and memory immune responses. Vaccines mimic the pathogen-induced immune response when administered by the systemic or mucosal route. In subcutaneous/intramuscular vaccination, Ag interaction occurs at the level of the skin epidermis (**A**). Then, the antigen is taken up by Langerhans dendritic cells, which migrate to nearby axial or cervical lymph nodes to activate naive T cells, resulting in the production of cytokines and chemokines, expression of co-stimulatory molecules, all of which will influence the nature and magnitude of the innate, secondary antibody responses (IgG, IgA), and peripheral CD4+T cell immune responses. The mucosal immune system includes the inductive sites, Peyer’s patches, mesenteric lymph nodes in GALT (small intestine) or lymphoid follicle-associated in BALT (bronchial-alveolar), and the effector sites (lamina propia) (**B**,**C**). In mucosal vaccination (oral, nasal), two main outcomes are possible: a mucosal and a systemic immune response leading to activation and differentiation into different CD4+, CD8+ T cells, and B cell subsets (**B**,**C**). The induction of cytokines, chemokines, and the expression of homing receptors populate the effector sites (lamina propia) in the different mucosal tissues (common mucous system), resulting in a long-lasting protective memory immune response. The induction of antibodies with enhanced functional properties (neutralizing and memory) as secretory IgA (sIgA). Cytokines such as TGF-β promote isotype switching of IgG class antibodies. Cytokines such as IL10 and IL-17 maintain homeostasis of the host response.

Vaccination is based on two features: (a) the induction of specific immunological memory and effector responses based on the priming and activation of B and T cells and (b) the induction of the inflammatory response to link the innate and adaptive immune response [1,2,3] at the systemic level after intramuscular immunization (Figure 3A).

Therefore, the question that has been the subject of intense research for decades, what is the best formulation of a vaccine candidate, to mount significant long-lived cellular effector and central memory to enable the efficacy of secondary specific IgG and secretory IgA antibody responses [49,66,67,68,69,70,71,72]. The magnitude of the response depends on the route of immunization, adjuvants, and prime-boost protocols (Figure 3B,C). Vaccines mimic airborne pathogen entry via the mucosal route by inducing a local inflammatory pathway following mucosal exposure to alveolar macrophages [6,19,34,50,51] (Figure 3B,C). The vaccine candidate interacts with the PRRS on the antigen-presenting cells and, in combination with the adjuvant, endocytosis favors cytosolic receptors and triggers one of these signaling pathways. In addition, STING, a stimulator of IFN genes, is an endoplasmic reticulum (ER)-associated four-pass transmembrane (TM) protein encoded by the STING1 gene (formerly TMEM173). STING responds to external ligands (bacterial CDNs) and second messengers (2′3′-cyclic GMP-AMP, 2′-3′-cGAMP) produced by the enzyme cGAMP synthase (cGAS). Once activated by bacterial CDNs, STING engages TANK-binding kinase-1 (TBK1) and IFN regulatory factor-3 (IRFs), which are key downstream effectors [73,74,75].

In addition, one of the complexities of vaccine development is that the molecular mechanism of pathogenicity and the immune mechanism of vaccine-induced protection remain poorly understood. In addition, most of these studies are based on the mouse model. Therefore, there is still a long way to go before subunit vaccines are used in clinical practice. Single-cell multi-omics technologies (transcriptomics, genomics, proteomics, metagenomics, metabolomics, and lipidomics) are making it possible to dissect and elucidate the understanding of this mechanism in the host–pathogen interaction with implications for the immune response [76,77]. Induced protection is one of the issues that can demonstrate the feasibility of therapeutic and prophylactic immunization. An epitope vaccine is a promising option for protection against *H. pylori* because, after vaccination, the antigen-specific response is predominantly polarized towards a Th2-type response, with the production of cytokines that can inhibit the activation of Th1 cells and macrophages and the production of pro-inflammatory cytokines [78]. In this study, the multi-epitope vaccine combines the cholera toxin B subunit (CTB), two antigenic fragments of the *H. pylori* urease I subunit (UreI20-29, UreI98-107), and four antigenic fragments of the *H. pylori* urease B subunit (UreB12-23, UreB229-251, UreB327-400, UreB515-561), resulting in recombinant CTB-UreI-UreB (BIB) [79,80]. The mechanism of protection, evaluated in the Balb/c mouse model and compared with recombinant CTB (rCTB) or PBS, results in the induction of specific serum IgA and mucosal sIgA and a mixed Th1/Th2/Th17 cell response. This multi-epitope vaccine may be a promising vaccine candidate to help control *H. pylori* infection [81]. Moreover, other multivalent *H. pylori* subunit vaccine candidates are composed of three commonly used *H. pylori* antigens, neutrophil-activating protein (NAP), urease subunit A (UreA), and subunit B (UreB), with the mucosal adjuvant, the heat-labile toxin (a mutant, dmLT) from *Escherichia coli* (*E. coli*). This vaccine induced a significant reduction in gastric bacterial colonization (at both 2 and 8 weeks post-immunization). There was a marked increase in serum antigen-specific IgG responses and mucosal IgA responses, as well as the induction of Th1/Th17 immune responses. The authors proposed that the overall effect of the vaccine candidate would be to overcome the immune evasion mechanism of *H. pylori* and to restore the suppression of Th2 immune responses with the induction of a strong humoral immune response, while the synergistic role of these CD4+ T cells with specific antibodies may favor the elimination of *H. pylori* [79,80,81] (Figure 2 and Figure 3B).

Furthermore, vaccines can also induce protective immune responses through a cell-targeting surface delivery system. For example, lactic acid bacteria such as *L. lactis*, called plSAM, support vaccine antigens and stimulate effective immune responses in the gastrointestinal tract. An M-cell-targeting recombinant *L. lactis* vaccine LL-plSAM-FVpE uses the plSAM surface display system. The recombinant *L. lactis* vaccine LL-plSAM-FVpE secretes and expresses the SAM-FVpE protein and displays it on the bacterial surface. As a result, L-plSAM-FVpE has enhanced M cell targeting properties and could be a promising vaccine candidate to control *H. pylori* infection [81,82,83,84]. In addition, LL-plSAM-FVpE has excellent M-cell-targeting properties to promote phagocytosis and the trafficking of the SAM-FVpE antigen by gastrointestinal M cells [81] (Figure 3C). More importantly, oral immunization with LL-plSAM-FVpE or SAM-FVpE plus PA can stimulate IgG and IgA antibodies and CD4+ T cell immune responses against four virulence factors of *H. pylori* (urease, CagA, VacA and NAP), thus providing protective immunity against *H. pylori* infection in mice. Furthermore, bacterial membrane vesicles (MVs) are nanoscale vesicular structures with diameters ranging from 20 to 400 nm. MVs contain bacterial lipids, proteins, and often nucleic acids from Gram-positive and Gram-negative bacteria, such as *Salmonella typhimurium* (*S. typhimurium)*, and efficiently stimulate the host immune response against bacterial infections (Figure 1 and Figure 2). The activated dendritic cells (DCs) contain major antigens (Ags) recognized by *Salmonella-specific* B cells and CD4+ T cells and protect against *S. typhimurium* challenges in a mouse model [85]. However, one of the disadvantages of MVs is that they can be harmful due to the carriage of toxic components, such as lipopolysaccharides, hemolysis and enzymes, which limits their clinical application. Therefore, it is a priority for engineered MVs to reduce MV toxicity, enhance vesicle immunogenicity, and increase vesicle production to have broad applications in vaccine design, vaccine delivery vesicles, and drug delivery systems [83,84,85]. MVs combined with nanoparticles (NPs) improve the homogeneity, stability, and function of MVs and have great potential for immunotherapeutic applications such as bacterial vaccines and cancer immunotherapy [85]. Of note is the ability of *S. typhimurium* MV to encapsulate an exogenous model antigen and stimulate antigen-specific CD4+ and CD8+ T cell responses, demonstrating the immunogenicity and in vitro immune response of MVs and their potential utility as a vaccine platform [86,87,88]. On the other hand, the spike protein contains several epitopes that play a role in the induction of neutralizing antibodies (mAbs) and protective immunity, in addition to T-cell responses against SARS-CoV-2. Furthermore, Zhang et al. 2022 [65] have reported a vaccine with immunogenic potential consisting of a mosaic trimeric form of the Spike receptor binding domain (mos-tri-RBD) carrying key mutations from Omicron and other circulating variants. Animals vaccinated with the spike-binding protein elicited immunoglobulin G (IgG) and A (IgA) antibodies in the nose and lungs, while animals vaccinated intramuscularly elicited a serum IgG response [86]. In addition, using a vaccination protocol and the mouse model, they showed that a two-dose regimen of FLUCOV-10 elicited a robust humoral immune response of IgG antibodies, neutralizing antibodies, and antigen-specific cellular protective immune responses against both homo- and heterologous influenza strains and SARS-CoV-2 [87] (Figure 2 and Figure 3B). Therefore, the main goal of vaccines against SARS-CoV2 (100 to 150 vaccines) is to reduce morbidity and mortality. It is also a goal to develop herd immunity in populations, including immunocompromised individuals [88]. All licensed COVID-19 vaccines and the vast majority of COVID-19 vaccines in development are administered intramuscularly to induce systemic immunity [89,90,91,92,93]. Of note, 8 of the 112 vaccines in clinical development are administered intranasally [90,93].

In general, the ingredients of the vaccine candidate for the induction of protective and memory immune responses could be outlined as follows: an antigen carrier an antigen (mostly are negatively charged), and the adjuvants (cationic, anionic) [94,95,96,97,98,99]. Moreover, the adjuvants used as delivery systems are mineral salts, aluminum salts, and calcium phosphate. Adjuvants are used as immunomodulators or immunopotentiators (monophosphoryl lipid A (MPLA, LPS, flagellin, CpG, poly IC, QS21; ISCOMs). The ingredients also comprise mucosal adjuvants (bacterial toxins) and systemic adjuvants such as AS01, AS03, and AS04. The most optimal adjuvants are type I IFNs, antimicrobial peptides, chemokines, and the use of nanoparticles [94,95,96,97,98,99]. In addition, there is also adjuvants as stimulators of the humoral response (e.g., inflammasome activator) and a stimulator of the cellular response (e.g., Toll-like Receptors, TLRs ligand).

### How Do Adjuvants Enhance and Broaden the Induction of Protective and Even Memory Immune Responses?

It is proposed that adjuvants, particularly mucosal adjuvants, link innate and adaptive immune responses and direct them towards B and T cellular protective and memory immune responses. The adjuvants act as pathogen-associated patterns (PAMPs) that interact with patterns of receptor recognition (PRRs) on APCs. They also act as agonists for TLR ligands. Each TLR has a leucine-rich repeat (LRR) that mediates PAMP/DAMP recognition and a TIR domain that provides downstream signaling and which initiates an inflammatory response. In addition, adjuvants can promote the production of pro-inflammatory cytokines and directly activate antigen presenting cells (APCs). MPL can enhance antibody responses in humans and cytotoxic CD8+ T lymphocyte (CTL) responses in mice through modified antigen cross-presentation by cDCs (QS-21) (Figure 2). Indeed, vaccine candidates combined with adjuvants shape the response of dendritic cells by enhancing their activation and recruitment to the inducer lymph node sites for T and B cell activation [4,99,100,101]. Moreover, AS01, Monophosphoryl Lipids A (MPL), an adjuvant system, is included in several vaccine formulations. It is a liposome-based adjuvant containing two immune stimulants. MPL is a detoxified lipid from Salmonella Minnesota LPS and a Toll-like receptor 4 (TLR4) agonist). QS-21 is a saponin molecule extracted from the bark of the *Quillaja saponaria* tree, a liposomal formulation that has recently been used in the formulation of vaccines against intracellular pathogens such as M. tuberculosis (tuberculosis), varicella zoster virus (VSV) (varicella), human immunodeficiency virus (HIV), and *Plasmodium falciparum* (malaria) [102,103,104].

## 5. Conclusions and Remarks

The host–pathogen interaction interface represents a rich source of molecular components to advance the dissection of the mechanism of immune system priming in systemic and mucosal compartments. The components of vaccine formulation play a role, along with other factors, such as the route of immunization and prime-boost protocols in the effective induction of B and T cells and protective and memory immune responses. Over the past decade, significant advances in adjuvants and antigen delivery systems have improved vaccine efficacy and have increased the potential for better local targeting. In vivo studies (mouse model) by our group have evaluated the potential adjuvant properties of Bacillus thuringiensis Cry toxins, such as type I IFNs in the mouse model, with promising results. In addition, there are numerous studies of and reports on the mouse model and clinical trials of various microbial candidates against a range of infectious diseases. However, it is essential to continue to dissect the molecular mechanism of pathogenicity for the design of vaccines and immunotherapies.

The COVID-19 pandemic and the emergence of multidrug resistance in microbes have made it an urgent priority to continue research to identify novel vaccine formulations that induce effective humoral and cellular immune responses with long-lasting B and CD4+ T cell responses. Translational science studies in animal, in vitro, and ex vivo models assess vaccine candidate efficacy and quantify the kinetics of vaccine immunity in the clearance of pathogens. In addition, there is an attempt to discover the correlates of protection in the form of functional markers or biomarkers to quantify vaccine kinetics. The most ad hoc vaccine formulation today is the one that can induce trained immunity and long-lasting central memory. The ideal formulation is whole, killed, or attenuated pathogens without adjuvants (first-generation vaccines). However, current vaccine formulations are based on microbial components and adjuvant(s), and some are developed in delivery systems such as exosomes, liposomes, membrane vesicles, and nanoparticles. Furthermore, for second- and third-generation vaccines, a systematic pipeline strategy using bioinformatics and omics technologies combined with AI is needed to accelerate and integrate the knowledge gained from both and extrapolate it to the different emerging threats.
